# Bioinspired figure-ground discrimination via visual motion smoothing

**DOI:** 10.1371/journal.pcbi.1011077

**Published:** 2023-04-21

**Authors:** Zhihua Wu, Aike Guo

**Affiliations:** 1 School of Life Sciences, Shanghai University, Shanghai, China; 2 State Key Laboratory of Brain and Cognitive Science, Institute of Biophysics, Chinese Academy of Sciences, Beijing, China; 3 International Academic Center of Complex Systems, Advanced Institute of Natural Sciences, Beijing Normal University at Zhuhai, Zhuhai, Guangdong, China; 4 University of Chinese Academy of Sciences, Beijing, China; Northeastern University, UNITED STATES

## Abstract

Flies detect and track moving targets among visual clutter, and this process mainly relies on visual motion. Visual motion is analyzed or computed with the pathway from the retina to T4/T5 cells. The computation of local directional motion was formulated as an elementary movement detector (EMD) model more than half a century ago. Solving target detection or figure-ground discrimination problems can be equivalent to extracting boundaries between a target and the background based on the motion discontinuities in the output of a retinotopic array of EMDs. Individual EMDs cannot measure true velocities, however, due to their sensitivity to pattern properties such as luminance contrast and spatial frequency content. It remains unclear how local directional motion signals are further integrated to enable figure-ground discrimination. Here, we present a computational model inspired by fly motion vision. Simulations suggest that the heavily fluctuating output of an EMD array is naturally surmounted by a lobula network, which is hypothesized to be downstream of the local motion detectors and have parallel pathways with distinct directional selectivity. The lobula network carries out a spatiotemporal smoothing operation for visual motion, especially across time, enabling the segmentation of moving figures from the background. The model qualitatively reproduces experimental observations in the visually evoked response characteristics of one type of lobula columnar (LC) cell. The model is further shown to be robust to natural scene variability. Our results suggest that the lobula is involved in local motion-based target detection.

## Author summary

Flies are adept at target tracking during high-speed aerial maneuvers, although their brains are tiny. Along the pathway from the retina to the deep optic lobe, local directional motion at different positions within the visual field is first detected, based on which optic flow the fly experiences is then encoded. In contrast to extensive research on optic flow processing in the fly visual system, much less is known regarding how target detection is performed, even under the simple condition of a tethered fly. In this study, we built a fly inspired model to investigate how a textured figure is discriminated from a similarly textured background by relying on relative motion cues alone. We found that the secret involved in a network downstream of local motion detectors extracting the figure is to naturally exploit the temporal coherence of the moving figure. Without a response integration in time to a sequence of temporally ordered input frames, the downstream network fails to extract the figure, even if it has made full use of spatial coherence between adjacent pixels in individual frames. The modeling is helpful for understanding where and how target detection is performed in the visual system of flies. The result could also be inspiring for motion detection in machine vision systems that rely only on a single camera.

## Introduction

Flies are capable of discriminating an object from its background based on relative motion cues alone, despite the inherently low spatial resolution of their compound eyes. The neural mechanisms underlying motion detection have long aroused research interest. As early as approximately half a century ago, directionally selective motion-sensitive neurons were identified in the fly lobula plate via extracellular or intracellular recordings [1–4]. The direction selectivity of such large-field neurons emerges as a result of integrating the outputs of local motion detectors, i.e., T4/T5 cells [5,6]. Each large-field tangential cell acts like a matched filter tuned to extract particular optic flow patterns created during fly self-movement [7,8], guided course control and gaze stabilization [5].

In contrast to the extensive studies on optic flow processing in the fly visual system, much less is known about the neural mechanism underlying figure-ground discrimination or target detection. When a textured figure is presented against a similarly textured background, figure discrimination relies mainly upon the extraction of motion discontinuities [9,10].

At the retina level, the photoreceptors of male houseflies’ lovespots, i.e., the sex-specific frontal eye regions, have higher spatial resolutions and faster responses than female photoreceptors [11]. The enhanced optics and phototransduction contribute to the qualified retinal images needed by males, which exhibit superior performance in the rapid pursuit of their female conspecifics. In the optic ganglia of hoverflies, a class of neurons, known as ‘small target motion detectors’, respond highly selectively to very small targets [12–14]. Some types of responses occur without the need for relative motion cues [13], implying that other mechanisms different from figure-ground discrimination are involved [15]. In the lobula plates of blowflies, figure detection (FD) cells have been thought to be key neurons specialized in discriminating a moving target [16,17]. Inhibition from the so-called pool cells [10] with large receptive fields was suggested to be critical in causing the FD cells to develop selectivity for the target motion [18–19]. Given that FD cells all have different directional preferences and different receptive field properties, each individual FD cell should require a specifically customized pool cell. The neural computation underlying the accurate target extraction achieved via the FD and pool cells remains elusive.

Other cells involved in the processing of moving figures were recently identified in the lobula, the lobula plate’s neighbor in the lobula complex. The lobula houses dozens of types of visual projection neurons (VPNs), such as LC cells [20–23]. Many types of LC cells respond to motion stimuli [23–31]. Several types of LC cells respond to motion in ways that suggest that they are capable of discriminating between the background and figure-like foreground [26,30]. Some types of VPNs are involved in processing figures defined by higher-order theta motion [32]. Different from first-order (Fourier) motion, theta motion is a type of paradoxical motion in which the internal texture of the foreground figure moves coherently in the direction opposite of the figure itself [33–35]. These data imply the important role of the lobula VPNs in figure-ground discrimination.

Do the lobula circuits solve the figure-ground discrimination problem? Here, we address this question by developing a computational model inspired by fly motion vision. The model includes a layer of correlation-type elementary movement detectors (EMDs) [36] and a lobula network that is hypothesized to be downstream of the local motion detectors and have parallel pathways with distinct directional selectivity. Solving figure-ground discrimination is equivalent to extracting the boundaries between a target and its background based on discontinuities in the output of a retinotopic array of EMDs.

Individual EMDs cannot detect true velocities, however, because their responses depend not only on the target velocity but also on other properties of the target patterns, such as their luminance contrast and spatial frequency content [37,38]. Although discriminating a target by relying upon motion discontinuities in the intact response profile of an EMD array is impossible, our simulations suggest that the lobula network might naturally perform an efficient smoothing operation on the output of the EMD array across space and time. The spatiotemporal smoothing process is shown to enable robust figure-ground discrimination and the subsequent feature extraction step. The results suggest that the lobula is involved in local motion-based target detection.

## Results

### Fly inspired EMD-lobula network model

Our working hypothesis, upon which the model was established, assumes that the lobula obtains motion information from T4/T5 cells directly or indirectly via interneurons. Through these interneurons, the lobula is hypothesized in the present model to obtain access to directional motion signals from T4/T5 cells. Further discussion and predictions concerning the neuronal substrate of the model are provided in the Discussion section. Here, we focus on model simulations.

**Fig 1:**
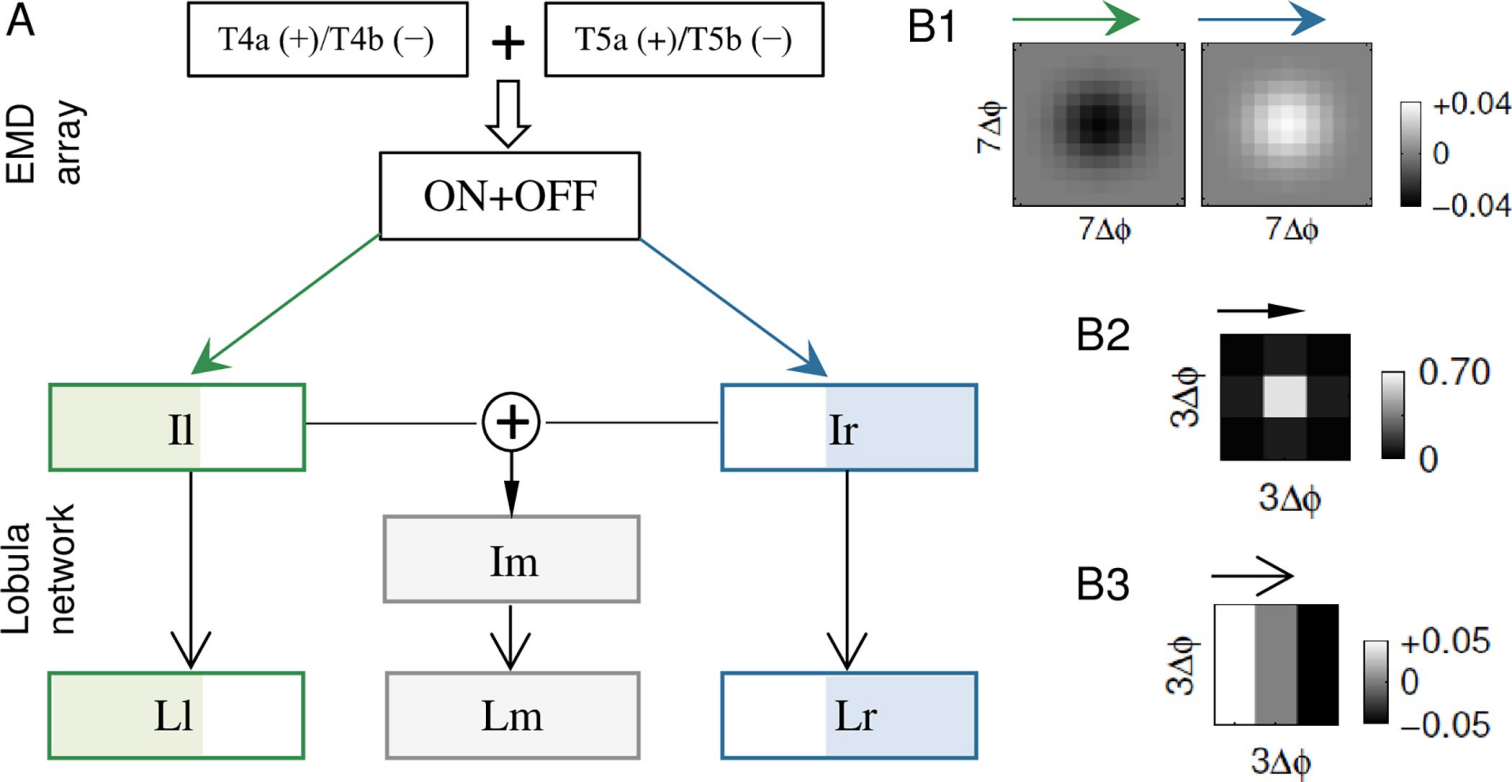
Schematic diagrams of the model. (**A**) EMD-lobula network. Individual EMD units comprise parallel ON and OFF pathways, which are exactly the same as shown in **Fig 4A** in the paper by Eichner et al. [39]. The output of the ON+OFF EMD array, as a retinotopic summation of two EMD arrays in the ON and OFF pathways, is projected to the Ir and Il modules. The outputs of Ir and Il are retinotopically added and projected to the Im module. The Lr, Ll, and Lm modules are postsynaptic to Ir, Il, and Im, respectively. Six lobular modules share the same array size as that of the ON+OFF EMD array. (**B**) Structures of example receptive fields of units belonging to Il (left panel in B1); Ir (right panel in B1); Im (B2); and Lr, Ll, and Lm (B3). Δϕ is the spatial interval between adjacent presynaptic units.

The model was developed as a three-layer feedforward network to simulate a circuit connecting T4/T5 cells and the lobula (**Fig 1**). No recurrent or feedback connections were considered for simplicity. The first layer is two arrays of EMDs that functionally simulate the pathway from the retina to T4 and T5 cells separately. We chose the 2-Quadrant-Detector model [39] to implement individual EMD units for reasons given in the Discussion section. The outputs of the two EMD arrays in the ON and OFF pathways were retinotopically added to form the final output of the ON+OFF EMD array, which was then projected to lobula network.

The second layer simulates interneurons that convey directional motion signals from T4/T5 cells to the lobula. This layer consists of three modules: Ir, Il, and Im, and “I”, “r”, “l”, and “m” stand for “Interneuron”, “right”, “left”, and “middle”, respectively. Neuron units in both Ir and Il received input from the ON+OFF EMD array but with opposite Gaussian receptive fields (**Fig 1A** and **1B1**). Spatial receptive field structures of the units in the Ir module were modeled as positive 2D Gaussian functions, whereas those of the units in the Il module utilized the same function but with a negative sign, making the two modules selective to opposite directional motions. Therefore, rightward local motion induced depolarization in the units in Ir but hyperpolarization in Il, and vice versa for leftward local motion. The outputs of the Ir and Il modules were retinotopically summed and projected to the Im module, making the units in Im have no directional selectivity. Spatial receptive field structures of the units in Im were modeled as positive 2D Gaussian functions with a side length of 3 lobula units (**Fig 1B2**).

The Ir, Il, and Im modules are projected to the Lr, Ll, and Lm modules in the last layer, respectively. “L”, “r”, “l”, and “m” stand for “Lobula”, “right”, “left”, and “middle”, respectively. Lr, Ll, and Lm inherited directional selectivity from their respective presynaptic modules. Their units were modeled as edge detectors in the lobula, whose receptive fields were set as Prewitt filters (‘Methods’) (**Fig 1B3**).

It is still unclear whether lobula neurons are spiking or graded potential neurons since their electrophysiological properties were probed by calcium imaging in most studies. Notably, using intracellular recordings, two types of lobula VPNs have been revealed to conduct signals by graded potentials. One is a type of lobula plate-lobula columnar neuron in calliphorid flies [40], and the other is a type of LC cell in *Drosophila* [25]. The model thus assumed that all neuron units in the six modules of the lobula network encoded inputs mainly by a graded shift of their membrane potentials for simplicity. By the term “membrane potential” throughout the paper, we mean a simulated value of the activity *V*(*t*) of the corresponding units according to their dynamics equation (see below).

We removed the spiking dynamics from leaky integrate-and-fire neuron model to simulate individual lobula units. The membrane potential *V*_i,j_(*t*) of a given unit located at (*i*, *j*) in a module obeyed the following integrate-and-nonspiking dynamics:

$$\tau_{m}\frac{dV_{i,j}(t)}{dt}=-V_{i,j}(t)+E_{\mathrm{Leak}}+RI_{i,j}^{\mathrm{syn}}$$ (1)

where *τ*_m_ is the membrane time constant, *E*_Leak_ is an equilibrium potential of leakage current, *R* is the total membrane resistance, and $I_{i,j}^{\mathrm{syn}}$ is the total synaptic input to the unit. When $I_{i,j}^{\mathrm{syn}}$ = 0, the potential relaxed exponentially with a constant *τ*_m_ to *E*_Leak_. Thus, *E*_Leak_ is also the resting potential of the model neuron. Because the electrotonic properties of most neurons in the fly’s optic lobe are unclear, we set *τ*_m_ = 5 ms and *E*_Leak_ = –50 mV by referring to the passive membrane properties of tangential cells in the lobula plate [41]. *R* was set as a dimensionless parameter, i.e., *R* = 1, such that the input $I_{i,j}^{\mathrm{syn}}$ was dimensionless for simplicity.

To determine the synaptic input $I_{i,j}^{\mathrm{syn}}$, the conductance matrix of a given postsynaptic module was calculated by convolving the receptive field function of the module with its presynaptic input matrix. The elements of the conductance matrices were disassembled into positive and negative components, representing excitatory and inhibitory synaptic conductances, respectively. The negative components were derived from either the null direction-selective output of the EMD array or the negative elements in the receptive field function of the postsynaptic module. Notably, synaptic conductances can never be negative in physiology. Inhibitory interneurons were not explicitly modeled in this study. We treated negative conductances as inhibitory conductances for simplicity. This treatment process aligns with the interpretation of negative conductances by Borst et al. [42], in which the negative outputs of local motion detectors were merged into inhibitory conductances.

The synaptic current for a given unit (*i*, *j*) was modeled as:

$$I_{i,j}^{\mathrm{syn}}(t)=\alpha [g_{i,j}^{+}(t)(E_{\mathrm{ext}}-V_{i,j}(t))+|g_{i,j}^{-}(t)|(E_{\mathrm{inh}}-V_{i,j}(t))]$$ (2)

where *E*_ext_ = 0 mV and *E*_inh_ = −80 mV are the reversal potentials of excitatory and inhibitory synaptic currents, respectively. $g_{i,j}^{+}(t)$ and $g_{i,j}^{-}(t)$ represent the positive and negative components of the input conductance, respectively. Parameter *α* represents a synaptic weight, which is hypothesized to be susceptible to adaptation and/or plasticity modulation (see ‘Methods’ for *α* value determination).

The EMD array and individual downstream modules shared the same array size, retinotopically covering a visual field of 180° in azimuth and 90° in elevation unless otherwise specified. Images of the visual inputs were sequentially presented to the EMD array, with each lasting 10 ms to simulate a 100 Hz refresh frequency. Under the speed condition of 33°/sec considered in our simulations, the displacement of input images was 0.33° within 10 ms, which is equivalent to one pixel, i.e., the smallest image element (‘Methods’). Therefore, a much higher refresh frequency was not necessary for the model stimuli. While the EMD array is a discrete system, the network of downstream modules is a continuous system whose neuron units are described by the ordinary differential Eq (1) with an integration time step 0.4 ms. By a “discrete system”, we mean here that the input of the EMD array was updated every 10 ms and remained unchanged within each update interval. The discrete updating process of the EMD input relieved the model of the burden of continuously receiving the same input every downstream module integration time step, yielding improved computational efficiency.

### Figure-ground discrimination downstream of the EMD array

**Fig 2:**
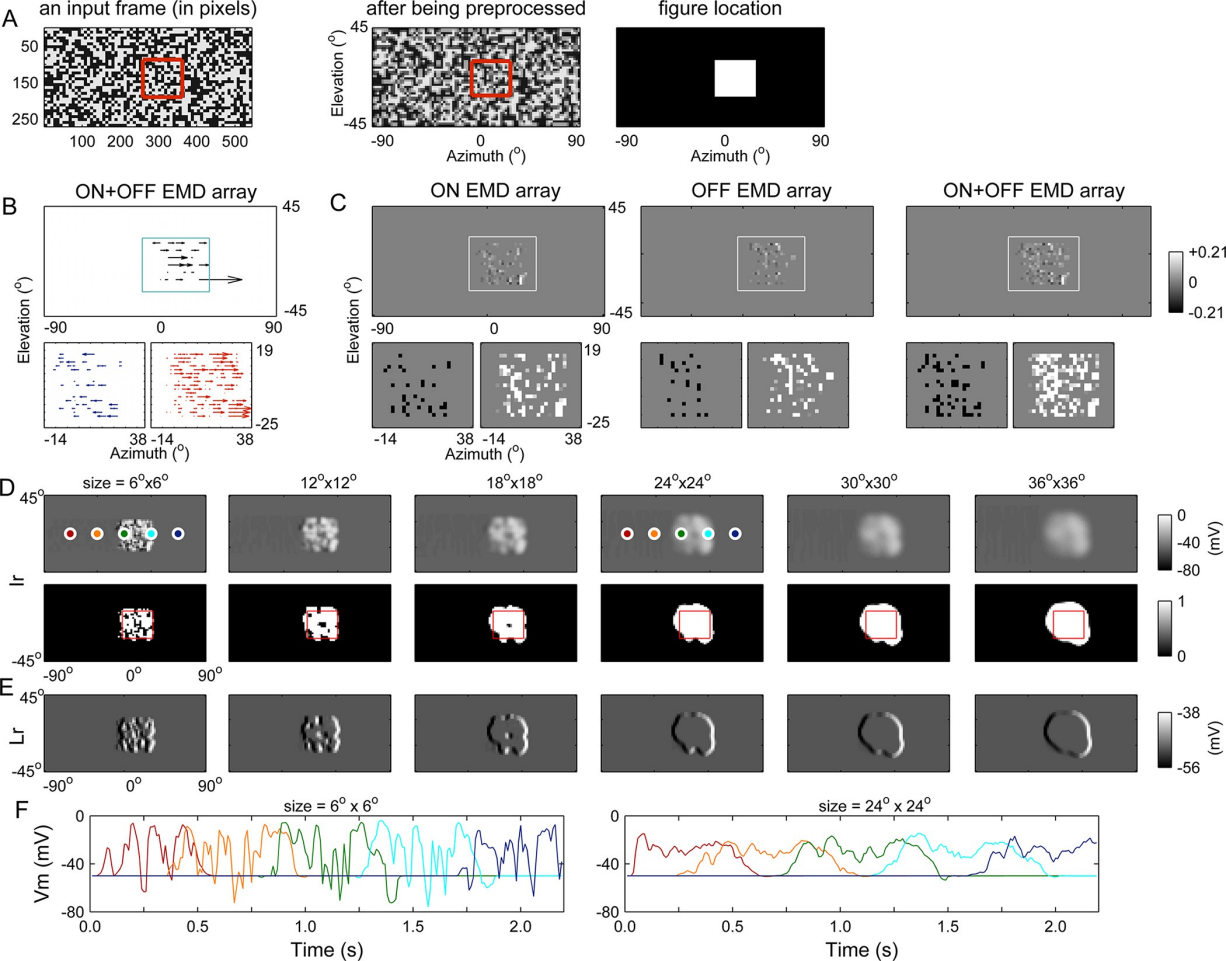
The lobula network solves figure-ground discrimination problems based on the noisy local motion measured by an EMD array. (**A**) One example of an input frame. The stimulus (80% contrast) consisted of a textured foreground figure (34° × 34°, in the red boundary) moving to the right at a speed of 66°/sec against a similarly textured background (stationary) (**S1 Video**). The red boundary was not present in the actual stimuli. Left: the original frame. Middle: the preprocessed image. Right: the foreground location (white area) and the background (black area). (**B**) Snapshot of the response profile of the ON+OFF EMD array. To examine the profile (top) at a higher resolution, the area outlined by the cyan boundary was zoomed in and disassembled into rightward (lower-right) and leftward (lower-left) components. (**C**) Same as (B) except that the EMD response profiles in the ON and OFF pathways were also displayed (upper row). All data were displayed as a grayscale matrix. The area in each panel outlined by the white boundary was magnified and disassembled into rightward (lower-right) and leftward (lower-left) components. Note: the element values were enlarged by 10-fold in the lower panels to facilitate an inspection of the difference between the numbers of elements of two components. (**D**) Foreground figures detected in the Ir module under different receptive field conditions (labeled at the top). Upper row: membrane potentials of the units in the Ir module. Lower row: output of the Ir module. Red boundaries indicate the figure’s locations at the corresponding instant in time. The membrane potential time courses of five units (marked by white circles) were further examined under two receptive field conditions in (F). (**E**) Membrane potentials of the units in the Lr module (simultaneously recorded with those in (D)). (**F**) Time courses of the membrane potentials of the five units marked in (D). The curves are color-coded according to the marking colors of the corresponding units in (D). The data in (A-E) are presented based on the input frame in (A). The synaptic weights of the projections between lobula modules were set as *α*^*lo*→*lo*^ = 2.

We first focused on the Ir and Lr modules to investigate whether they were sufficient for figure-ground discrimination (**Fig 1**). Individual EMD units detected local motion within their limited receptive fields, the array of which only provided a rough estimation of directional motion. To illustrate this, a randomly textured square moving on a similarly textured background was fed to the model (**S1 Video**) (**Fig 2A**). Although the figure kept moving to the right, leftward motion, i.e., negative components in the EMD output, was always detected (**Fig 2B and 2C**). The detected local motion presented a variety of vector magnitudes across space and time. This result is in accordance with the well-known fact that an EMD’s output does not exclusively depend on velocity but also on other properties of the stimulus patterns, such as the luminance contrast and spatial frequency content [37,38]. For periodic patterns such as a sine-wave grating, the EMD’s optimal response is determined by the temporal frequency (i.e., the ratio between the angular velocity and the spatial wavelength of the grating) rather than by the grating velocity alone. Therefore, discriminating a figure by relying upon the discontinuities of the visual motion measured by the EMD array is nearly impossible.

On the other hand, despite coexisting negative components, the rightward components always dominated the detected visual motion (**Fig 2B and 2C**), making the positive components of the EMD output dominate the receptive field of the downstream Ir module. We were inspired to think that a group of neighboring units in the Ir module, theoretically encoding the moving figure, should be simultaneously depolarized if their receptive fields are sufficiently large. We tested this idea by changing the receptive field size. Simulations showed that the units were depolarized to various extents depending on the sizes of their receptive fields (**Fig 2D**), in which the output of these units (lower row) was determined by applying the sigmoid activation function (see ‘Methods’ for its specific form) to their membrane potentials (upper row). Once the size reached approximately 24° × 24°, the figure was well separated or segmented from its background, confirming our hypothesis. A receptive field that was too large had no qualitative effect on the result. To examine the neural activities occurring during figure-ground segmentation, the time courses of the membrane potentials of five representative units in the Ir module obtained under two receptive field size conditions were also displayed (**Fig 2F**).

Simultaneously, the performance of the Lr module was also improved as the receptive field size of the Ir module increased. As an edge detector module, Lr not only detected figure edges but also discriminated the leading edge from the trailing edge (**Fig 2E**). The former was encoded by a group of depolarized units, and the latter was encoded by hyperpolarized units. Other resting (nonsignaling) Lr units were retinotopically matched to the figure surface or the stationary background.

**Fig 3:**
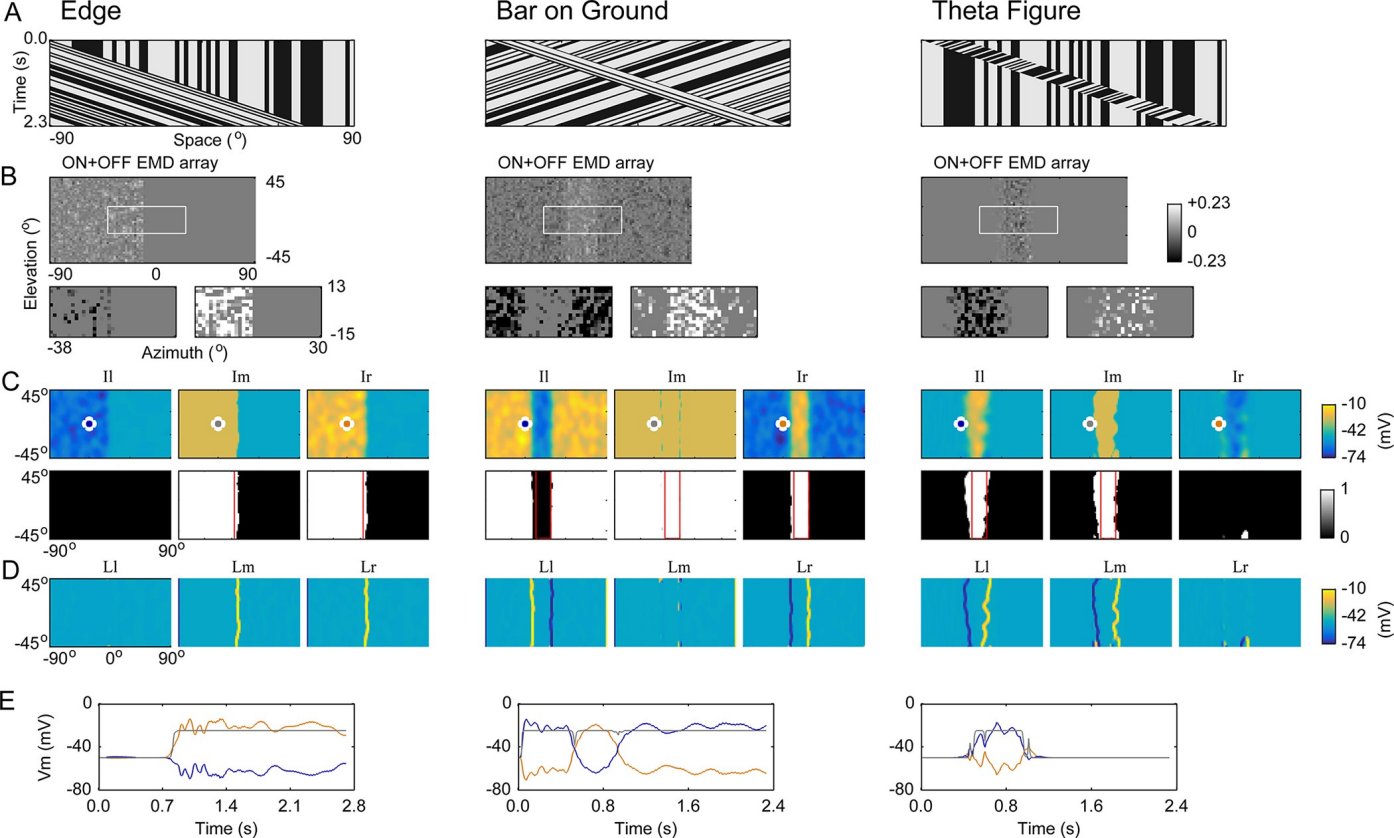
Individual lobula modules extract stimulus features depending on their receptive field structures and direction selectivity. Data are presented vertically according to their stimulus classes (indicated at the top) and shown based on the input frame at which the foreground figure passed the middle of the visual field. The receptive field of the units in Ir and Il on the EMD array was set as 26° × 26°. (A) Space-time plots of three classes of stimuli with a contrast level of 80% and a moving speed of 66°/sec. Each plot illustrates how the top row (horizontal axis) evolved with time (vertical axis). The top row was randomly selected from the rows of the first frame of stimulus images, so the row evolution characterizes the dynamic evolution of the stimulus. In ‘Bar on Ground’, the background was moving in the direction opposite of the bar (25° × 90°) at the same speed. In ‘Theta Figure’, the elements within the bar (25° × 90°) moved to the left, and the bar itself moved to the right at the same speed. (B) Output of the ON+OFF EMD array (upper row). The area outlined by the white boundary in each panel was magnified and disassembled into rightward (lower-right) and leftward (lower-left) motion components. The element values were enlarged by 10-fold in the lower panels to facilitate an inspection of the difference between the numbers of elements of the two components. (C) Membrane potentials (upper row) and corresponding outputs (lower row) of the units in the Ir, Il, and Im modules. Red boundaries indicate the actual locations of the moving foregrounds at the corresponding instant in time. The membrane potential time courses of the units (marked by white circles) were further examined in (E). (D) Membrane potentials of the units in the Lr, Ll, and Lm modules (simultaneously recorded with those in (C)). (E) Time courses of the membrane potentials of the units marked in (C). The curves are color-coded according to the marking colors of the corresponding units in (C).

Can the model be generalized to more complex tasks? We tested three other classes of stimuli: an edge moving on a stationary background (denoted as ‘Edge’), a bar moving on a reversely moving background (‘Bar on Ground’), and a bar with higher-order theta motion (‘Theta Figure’) (**Fig 3A**). In ‘Theta Figure’, black and white dots that were randomly distributed within the bar coherently moved in the opposite direction relative to the bar itself [33–35]. The results showed that although the EMD array provided considerably rough visual motion inputs to the lobula network (**Fig 3B**), the foreground figures were clearly segmented, and the figure edges were effectively extracted for all tested stimuli (**Fig 3C and 3D**).

The membrane potential of a representative unit in each of the Ir, Il, and Im modules illustrated the directional selectivity of the corresponding module at the single-unit level (**Fig 3E**). The preferred direction of Ir and Lr was right. The preferred direction of Il and Ll was left. The Im and Lm modules had no direction selectivity. The direction selectivity accounted for the distinct responses in three parallel pathways to a given stimulus. For instance, the Ir and Il (Lr and Ll) modules discriminated the figure (the figure edges) in the ‘Bar on Ground’ stimulus in opposite ways (**Fig 3C and 3D**, panels with ‘Bar on Ground’). For the ‘Theta Figure’ stimulus, Il (but not Ir) veritably discriminated the rightward-moving theta figure (**Fig 3C and 3D**, panels with ‘Theta Figure’). This is because the EMD was only able to detect coherent first-order motion within the figure, not the higher-order motion of the figure itself (**Fig 3B**, panels with ‘Theta Figure’).

The moving background in the ‘Bar on Ground’ stimulus disabled the Im module from discriminating the figure because the module was depolarized by both rightward and leftward motion. Consequently, its downstream module (Lm) failed to detect figure edges. The units of the Im and Lm modules were actually background-suppressed figure and edge detectors, respectively.

Our simulations indicated that neuronal hyperpolarization encodes information as important as neuronal depolarization. Compared with depolarization events, how can hyperpolarization events be conveyed to postsynaptic neurons in the lobula to ensure that downstream neurons are correctly informed in the context of figure-ground discrimination? We speculate that the spontaneous baseline activities of related lobula neurons or inhibitory interneurons may be involved in the solution.

Taken together, our simulations suggest that the lobula circuit can solve figure-ground discrimination problems based on roughly measured visual motion alone. The solution involves three parallel pathways, which process rough visual motion according to distinct directional selectivity.

### Spatiotemporal visual motion smoothing via the lobula network

Since the spatial receptive field structure of the neurons postsynaptic to the EMD array was described by a 2D Gaussian filter (‘Methods’), can we attribute the success of figure-ground discrimination to the Gaussian smoothing of the EMD array’s output by downstream modules? To answer this question, we dissected the motion signal processing procedure by focusing on the EMD-Ir part of the model.

The signals at each stage were extracted and transformed to a series of binary images by setting a 50% maximum as the segmentation threshold, so that we were able to quantitatively evaluate figure-ground discrimination effect. The segmented foreground and background pixels were represented by white and black pixels, respectively. Given that the stimuli were simple, we manually annotated every moving foreground bar as a ground truth. We used F-measure to evaluate the degree of correctness exhibited by figure-ground discrimination at each stage because the F-measure is a commonly used metric for assessing model performance in a two-class classification problem. The F-measure metric is defined as the harmonic mean between two other metrics called *precision* and *recall*:

$$F‐measure=\frac{2}{\frac{1}{precision}+\frac{1}{recall}}$$ (3)

where *precision* is the fraction of correctly classified foreground pixels among all classified foreground pixels, and *recall* is the fraction of correctly classified foreground pixels among all foreground pixels in ground truth (see ‘Methods’). The F-measure is a value between 0.0 for the worst case (failed figure-ground discrimination) and 1.0 for the best case (perfect discrimination).

**Fig 4:**
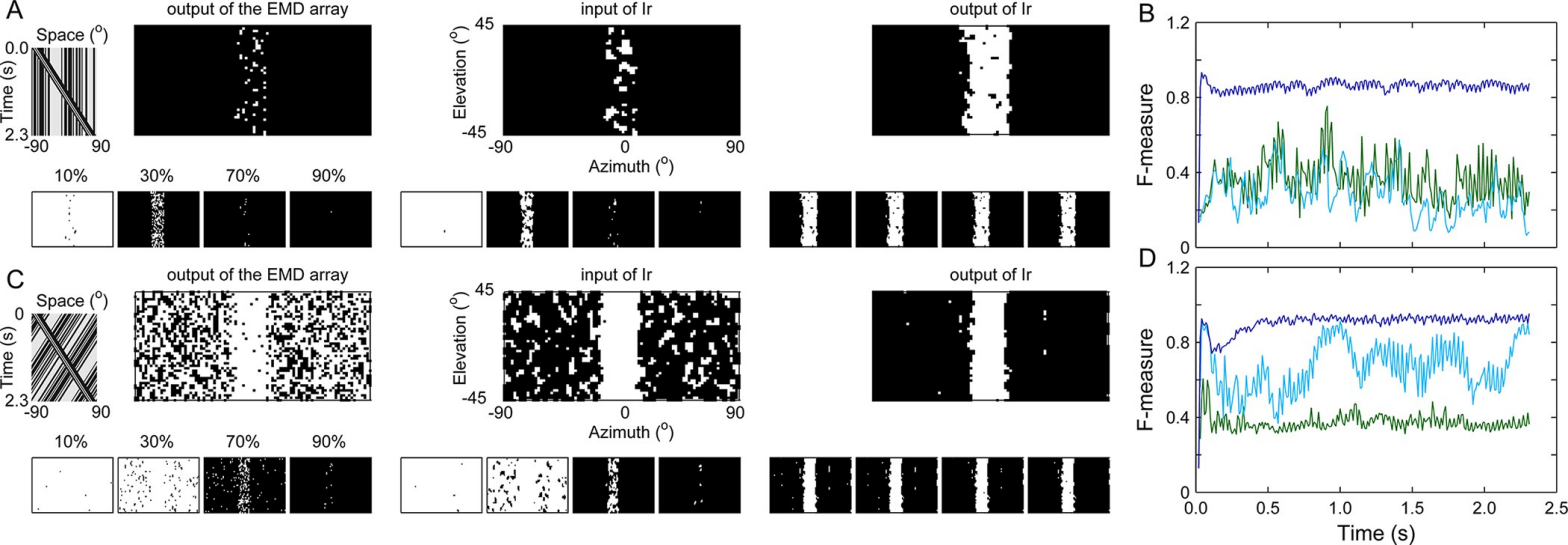
Dissecting the figure-ground discrimination process in the pathway from the EMD array to the Ir module. (**A**) Snapshots of the segmented foreground (white area) and background (black area) at three stages: the output of the EMD array (left column), the input of the Ir module (middle column), and the output of the Ir module (right column). The segmentation threshold was set as 50% maximum (top row) or 10%, 30%, 70%, and 90% maximum (bottom row). The stimulus was a textured 25° bar (80% contrast), which was moving against a similarly textured background at 66°/sec (upper leftmost: space-time plot). Data are presented based on the input frame at which the bar passed the middle of the visual field. (**B**) Instantaneous F-measure throughout the entire stimulus presentation period. It was evaluated by using a segmentation threshold of 50% maximum at three stages: the output of the EMD array (green curve), the input of the Ir module (light blue curve), and the output of the Ir module (dark blue curve). (**C**) and (**D**) are the same as (A) and (B), respectively, except that the stimulus background was moving in the direction opposite of the bar. For all simulations, the receptive field of the units in Ir and Il on the EMD array was set as 10°×10°.

**Fig 4C** shows the signals extracted at each stage of the EMD-Ir part when stimulated by the ‘Bar on Ground’ stimulus defined above. It is evident that the figure-ground discrimination effect was continuously improved from the output of the EMD array to the output of the Ir module via the input of the Ir module (**Fig 4C**, top row). The characteristic remained unchanged when the background was kept stationary (**Fig 4A**, top row). The data were also displayed separately using 4 other segmentation thresholds (10%, 30%, 70%, and 90% maximum) (**Fig 4A and 4C**, bottom rows). The figure-ground segmentation effect upstream of Ir was highly dependent on the threshold value. An ~30% threshold provided the best segmentation results under stationary background conditions, whereas an ~70% threshold was required under moving background conditions. In contrast, at the Ir level, the segmentation process was robust to changes in the threshold and stimulus type. Correspondingly, the F-measure was low at the output stage of the EMD array, and it was slightly decreased (under stationary background conditions) or randomly increased (under moving background conditions) when motion signals entered the Ir module (**Fig 4B and 4D**). In sharp contrast, the F-measure was robustly increased by the Ir module to values greater than 0.8 at the Ir output stage, indicating robust figure-ground discrimination. These results illustrate that along the processing flow of the model, figure-ground segmentation was not completely solved until the output stage of the Ir module. In other words, we cannot attribute the success of figure-ground discrimination to the Gaussian smoothing of the EMD array’s output by downstream modules.

**Fig 5:**
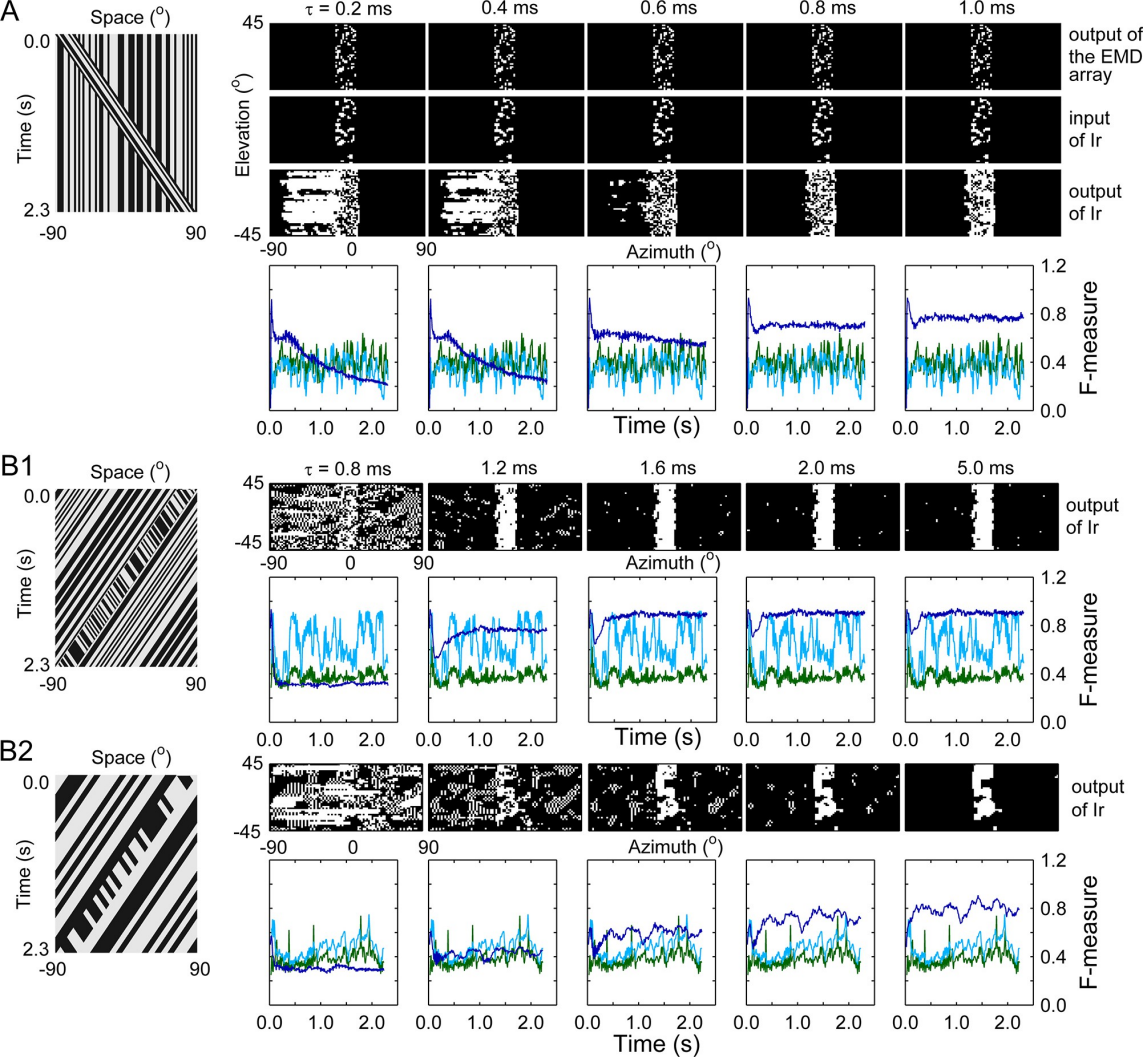
Effect of the membrane time constant *τ*_m_ on figure-ground discrimination. (**A**) Figure-ground segmentation effect achieved with the same stimulus as that used in Fig 4A. Leftmost panel: space-time plot of the stimulus. The 1st to 3rd rows: snapshots of the segmented foreground (white area) and background (black area) at three stages (the output of the EMD array (1st row), the input of the Ir module (2nd row), and the output of the Ir module (3rd row)). The snapshots are presented based on the input frame at which the bar passed the middle of the visual field. The segmentation threshold was set as 50% maximum. The corresponding *τ*_m_ value is indicated at the top. The 4th row: instantaneous F-measures throughout the entire stimulus process evaluated at the output of the EMD array (green curve), the input of the Ir module (light blue curve), and the output of the Ir module (dark blue curve). (**B1**) Same as (A), except the stimulus was replaced by ‘Theta Figure in Moving Background’ (leftmost panel). The stimulus featured a background moving to the left at 66°/sec. The bar shared exactly the same motion as the background, whereas the elements within the bar coherently moved to the right at 66°/sec. (**B2**) Same as (B1), except the dot size of the stimulus was changed from 2.6° to 7.9°.

The above dissection of the signal processing procedure indicates that smoothing visual motion based only on spatial features, as reflected at the input stage of the Ir module, was not sufficient for achieving a robust figure-ground discrimination effect. Further temporal integration of the visual motion in Ir was critically needed. To probe the temporal integration property, we repeated the simulation in **Fig 4A** by changing the membrane time constant *τ*_m_ of the Ir module (see Eq (1)) (**Fig 5A**). Figure-ground segmentation failed if the *τ*_m_ value was less than 0.6 ms, as shown in both the output snapshots of the Ir module (**Fig 5A**, 3rd row) and the F-measure curves (**Fig 5A**, 4th row). Once the *τ*_m_ value reached 0.8 ms, a robust segmentation effect appeared. These values (< 1 ms) actually fall within the range of equalization time constants, which are shorter than membrane time constants of measured neurons in the insect optic lobe [41, 43].

To scrutinize whether the *τ*_m_ threshold depends on the stimulus type, we repeated the simulation with a much more complex stimulus called ‘Theta Figure in Moving Background’ below (**S2 Video**) (**Fig 5B1**, leftmost panel). Different from ‘Theta Figure’ (**Fig 3A**, rightmost panel), in the new stimulus, the background moved together with the bar figure. Specifically, both the bar and its background moved to the left at the same speed, and elements within the bar coherently moved to the right at the same speed as the bar and background. Simulations revealed that a *τ*_m_ value of 0.8 ms failed to produce a successful segmentation result, and the best segmentation effect did not appear unless the *τ*_m_ value reached 1.6 ms (**Fig 5B1**). We next changed the dot size of the new stimulus’s texture from 2.6° to 7.9° and repeated the simulation process once again (**S3 Video** and **Fig 5B2**, leftmost panel). Compared with the result obtained under the smaller dot size condition, a much larger *τ*_m_ value was required to achieve a reasonable segmentation effect for larger dot size stimuli (**Fig 5B2**). Taken together, for all stimuli used in the study, a time constant of 5 ms was sufficiently large to allow the lobula units to perform a robust temporal integration of the visual motion.

The above results indicate that almost any neuron is competent for the required temporal integration, as long as the neuron possesses memory of the inputs for a short period of time (>5 ms in the case of our model parameters). The Gaussian smoothing of the EMD array’s output within the receptive fields of downstream lobula units strengthened the dominant components of the output to various extents. The effect of this strengthening operation at a given instant depended only on the EMD array’s output at that instant, which caused the fluctuating F-measures, as shown by the simulations (light blue curves in **Figs 4 and 5**). After the strengthened EMD output entered the lobula network, the present as well as past output of the EMD array was integrated in the lobula units’ responses. This is because the lobula units were essentially individual dynamic systems, whose responses depended not only on the present inputs but also on the past inputs. The temporal integration of the inputs accounts for the robust and boosted F-measure (dark blue curves in **Figs 4 and 5**).

In summary, the key to the solution of figure-ground discrimination is that the noisy output of the EMD array was spatially and temporally integrated and, thus, smoothed out by the downstream lobula units.

### Effects of the receptive field size and stimulus parameters on figure-ground discrimination

Parameters such as the dot size and bar speed affected the output of the EMD array due to the pattern-dependent responses of the detectors [37,38]. Furthermore, the sizes of the receptive fields of the Ir and Il modules could affect the processing of the EMD responses in the lobula network. To quantitatively explore the effects of these factors on model performance, we focused on the EMD-Ir part in this section.

**Fig 6:**
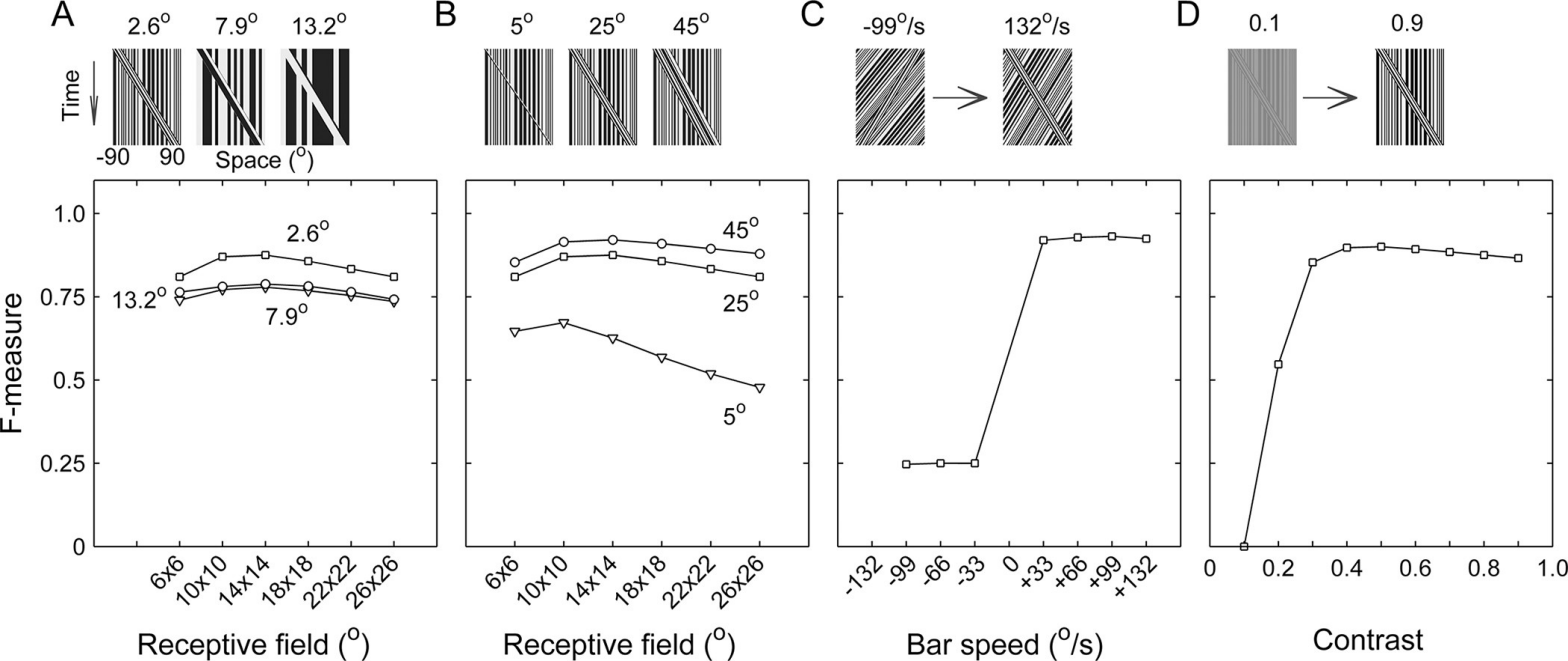
Effects of the receptive field size and stimulus parameters on figure-ground discrimination. Unless specified below, the receptive field size of the lobula units on the EMD array was set as 14° × 14°, and a moving bar (25° width; 80% contrast; 2.6° dot resolution; 66°/sec speed) with a stationary background was used as the stimulus. The F-measures were averaged across the input frames (with the initial 50 frames excluded as transient frames) at the output stage of the Ir module. (**A-B**) Effect of the receptive field size on the averaged F-measure under different stimulus conditions. In panel (A), three dot resolutions, as specified at the top of each space-time plot, were tested: 2.6° (squares), 7.9° (triangles), and 13.2° (circles). In panel (B), three bar widths, as specified at the top, were tested: 5° (triangles), 25° (squares), and 45° (circles). (**C-D**) Effects of the stimulus velocity (C) and contrast (D) on the averaged F-measure. In panel (C), the background was not stationary but rather moving at −132°/sec, and the bar’s velocity was varied as specified along the abscissa axis.

We first ran the model with various dot size and bar width combinations for the ‘Bar on Ground’ stimulus, which effectively changed the spatial frequency distribution of the input pattern. Except for the case with a very narrow bar (5° wide), most tested dot sizes and bar widths led to high F-measures under different sizes for the receptive field of the Ir module (**Fig 6A and 6B**). Increasing the bar width improved the figure-ground discrimination effect, although the improvement lessened as the bar width continued to rise (**Fig 6B**). Varying the dot size alone had little influence on the F-measure, except for the case with a very small dot (2.6°), in which a higher F-measure was achieved (**Fig 6A**). The higher F-measure could have been caused by the much denser distribution of the EMD responses under the condition of a very small dot size.

Although an optimal receptive field size of approximately 12° × 12° was obtained, the role of the receptive field size in tuning the F-measure was weak. An exception was the case with a bar width of 5°, where the F-measure considerably decreased with the receptive field size. This is because the very narrow bar was discriminated as a much wider figure by the model with a receptive field size that was much larger than the bar width.

We next investigated the effect of a difference between the velocities of the figure and its background. By making the background move at a constant velocity and varying the bar velocity, simulations showed that figure-ground discrimination could be readily solved as long as the bar moved in the direction opposite of the background (**Fig 6C**). Poor performance arose once the bar shared the same direction with its background, even if their speeds were distinguishable. This could mainly be due to a nonmonotonic dependence of the EMD output on speed. For instance, under the stimulus condition of grating patterns with fixed spatial wavelengths, the response of a single EMD was unimodal rather than a linear function of the pattern velocity [37,38].

Finally, we varied the contrast level of the stimulus between trials. The figure was very effectively discriminated once the contrast was greater than or equal to 30% (**Fig 6D**). We used Michelson contrast for all stimuli in the study except for natural scene patterns, to which C_RMS_, i.e., the root mean square (RMS) contrast, was applied (‘Methods’). Lower contrasts considerably impaired the figure-ground discrimination process, which was qualitatively in accordance with the fact that the EMD response depended on the square of the pattern contrast [37,38].

### Comparison with LC cells

High specificity in the visual responses to moving stimuli has been reported in different types of LC cells when using two-photo calcium imaging [26,30,31]. An obvious question to ask is whether our model can account for the physiological measurements of LC cells.

**Fig 7:**
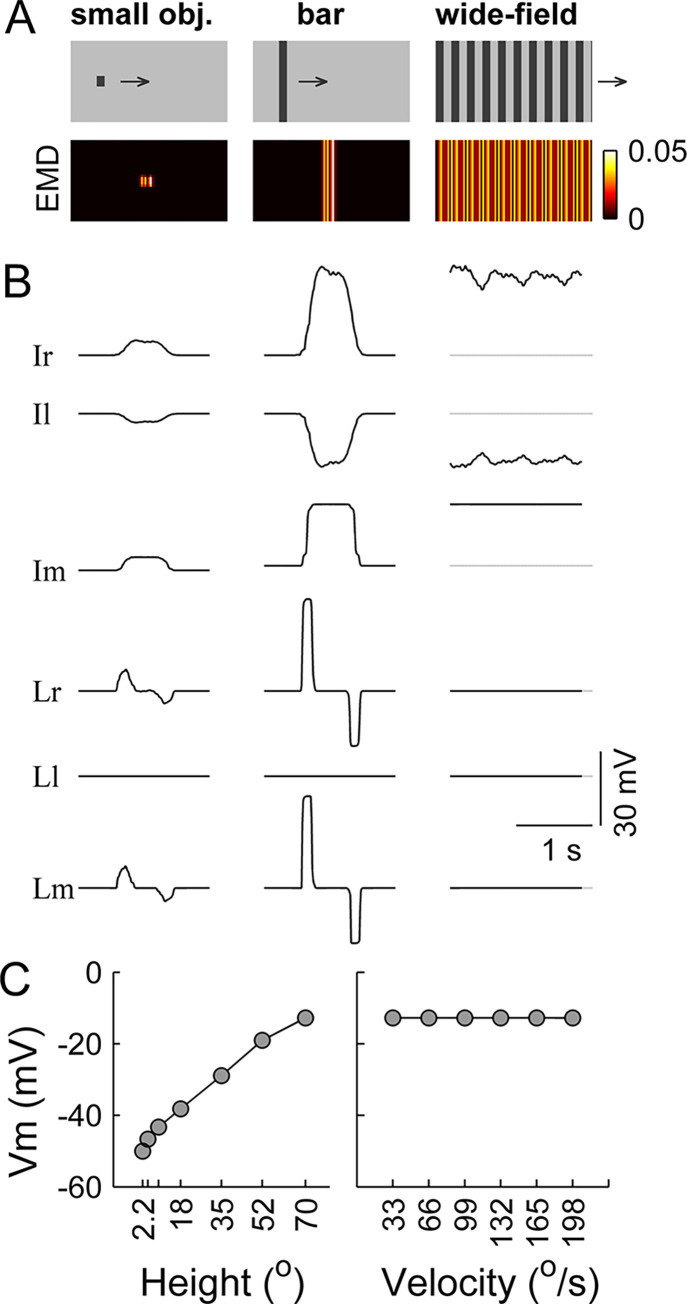
Stimulus-evoked responses at the single-unit level in the lobula network. (**A**) Three classes of stimuli (upper row, with their names indicated) and the corresponding output of the ON+OFF EMD array (lower row). The ‘small obj.’ and the ‘bar’ had figures with sizes of 8.9° × 8.9° and 8.9° × 70°, respectively, and the luminance levels of their foreground and background were set as 0.25 and 0.75, respectively. The ‘wide-field’ square grating had a spatial wavelength of 17.8° and a Michelson contrast of 50%, and its luminance was periodically set as 0.25 and 0.75. All the stimuli had 70° x 180° visual fields and moved rightward at 33°/sec. (**B**) Stimulus-evoked membrane potentials in the six lobula modules with the names indicated beside each row. Only the response of the centrally located unit in each module is displayed. Dashed lines indicate the resting membrane potentials. (**C**) Effects of the bar height and speed on the depolarization extent of the Lm module. Only the data of the centrally located unit are displayed. Left panel: the peak of the evoked membrane potential versus the bar height. Right panel: the peak of the evoked membrane potential versus the bar speed. For all simulations, the synaptic weights of the projections from the EMD array to the postsynaptic lobula modules were set as *α*^*EMD*→*lo*^ = 100.

We created the second type of visual stimuli (‘Methods’) to mimic those used by Städele et al. [30] (**Fig 7A**, top row). Specifically, three classes of stimuli were created: a dark small square moving over a light background (denoted as ‘small obj.’), a dark bar moving over a light background (denoted as ‘bar’), and a moving square grating that extended over the entire visual field (denoted as ‘wide-field’). All the stimuli had a visual field of 180° in azimuth and 70° in elevation and moved rightward. By feeding the stimuli to the model one by one, the simulations showed that the units in the Ir, Il, and Im modules responded to all 3 classes of stimuli, whereas the units in the Lr, Ll, and Lm modules responded only to the ‘bar’ or ‘small object’ (**Fig 7B**). The units in Ir and Il were depolarized by the motion in their preferred direction and hyperpolarized by the motion in their null direction. The preferred directions of Ir (rightward) and Il (leftward) were opposite to each other. The units in Lr and Ll were activated by the motion in their preferred direction and kept silent to the motion in their null direction. The units in Im and Lm had no direction selectivity. Taken together, only the units in the Lm module exhibited similar properties to the responses of a few LC types that were recorded previously [30,31]; specifically, they were suppressed by wide-field motion and were not directionally selective.

We repeated the simulation above by separately varying the bar height and speed. The depolarization of the Lm units increased with the bar height (**Fig 7C**, left panel) but was insensitive to the bar speed (**Fig 7C**, right panel). This property was qualitatively consistent with the response characteristic of LC15 [30,31]. The units in the Lm module are, thus, called LC15-like units below. It is worth noting that the hyperpolarization responses to the trailing edges of the ‘bar’ and ‘small object’ in LC15-like units were not reported in LC15 [30,31], which could be because calcium imaging-based recording is not sensitive to hyperpolarization activities.

**Fig 8:**
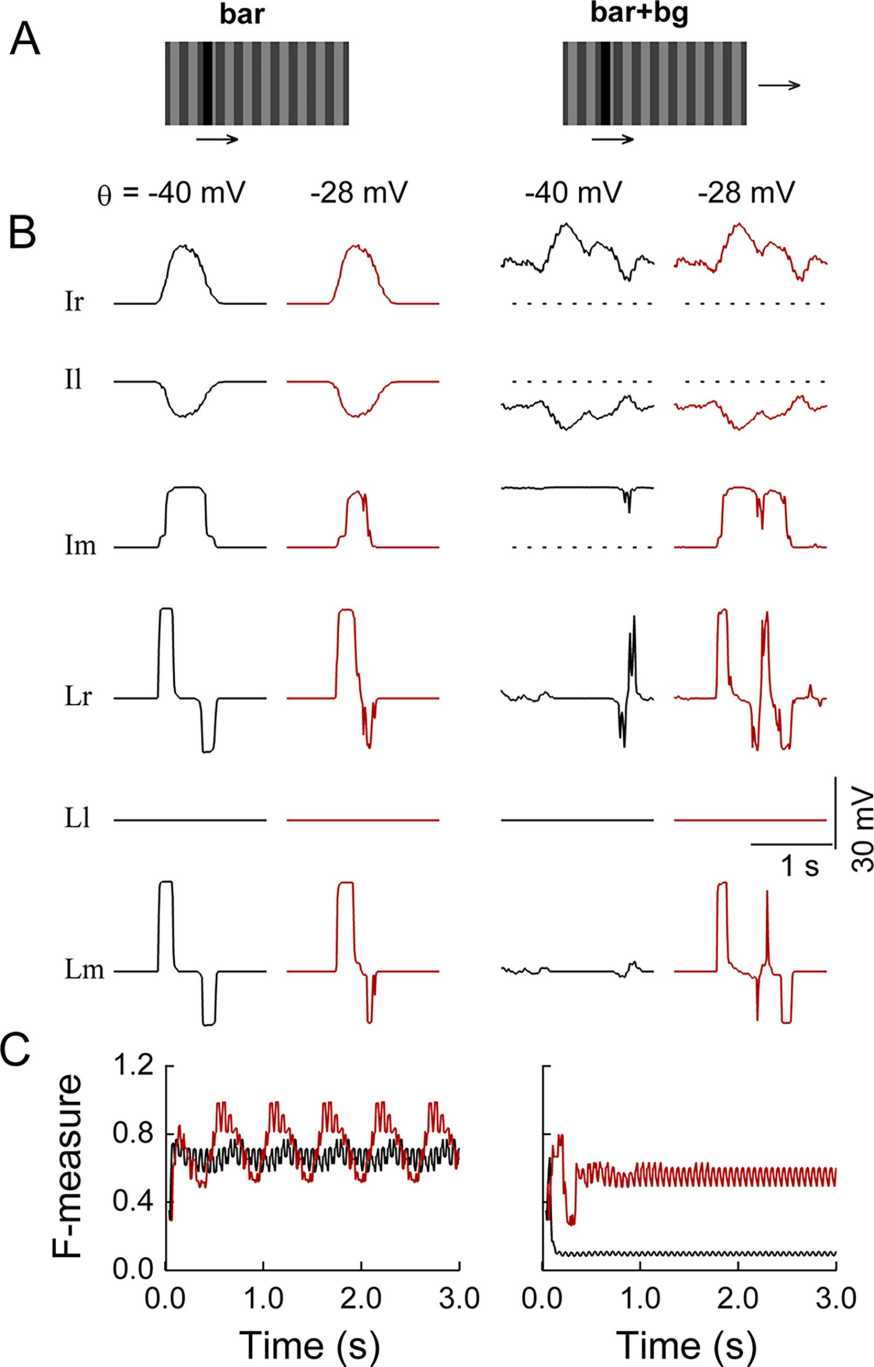
Effect of simulated octopaminergic modulation on the lobula network. (**A**) Two classes of stimuli. The dark bar (0 luminance; 8.9° width) moved rightward at a speed of 33°/sec over a background square grating with a Michelson contrast of 33% (the luminance was periodically set as 0.25 and 0.5; 17.8° spatial wavelength). The grating was either stationary (the ‘bar’ stimulus) or moving with the same velocity as the bar (the ‘bar+bg’ stimulus). The visual field was 70° × 180°. (**B**) Stimulus-evoked membrane potentials with (red curves) and without (black curves) octopaminergic modulation in the six lobula modules with the names indicated beside each row. Only the responses of the centrally located unit in each module were displayed. Octopaminergic modulation was realized by changing the half-activation voltage *θ* of the activation function of the Ir and Il modules from −40 mV to −28 mV. Dashed lines indicate the resting membrane potentials. (**C**) F-measure obtained under the corresponding stimulus condition. The F-measure with (red curves) and without (black curves) octopaminergic modulation was evaluated at the output stage of the Ir module during the continuous presentation of 300 input frames. For all simulations, the synaptic weights of the projections from the EMD array to the postsynaptic lobula modules were set as *α*^*EMD*→*lo*^ = 100.

We used similar stimuli to those employed in a previous study [30] (**Fig 8A**) to determine whether the LC15-like units are able to solve the figure-ground discrimination problem faced by the LC15 cells as presented by Städele et al. [30]. Specifically, two classes of stimuli were created, both of which had a dark bar moving over a low-contrast background grating. The square grating was either stationary (denoted as ‘bar’ in **Fig 8A**) or moving with the same velocity as the bar (denoted as ‘bar+bg’). The model failed to discriminate the dark bar once the background grating began to move in the same direction as the bar. This was illustrated by a remarkable decrease in the F-measure obtained with concomitant background motion (**Fig 8C**, black curve in left panel versus that in right panel). The evoked response to the bar in the LC15-like units was suppressed by the background motion, displaying a similar characteristic to LC15 except for the hyperpolarization part, which was not observed by Städele et al. [30] (**Fig 8B**, 1st versus 3rd column).

**Fig 9:**
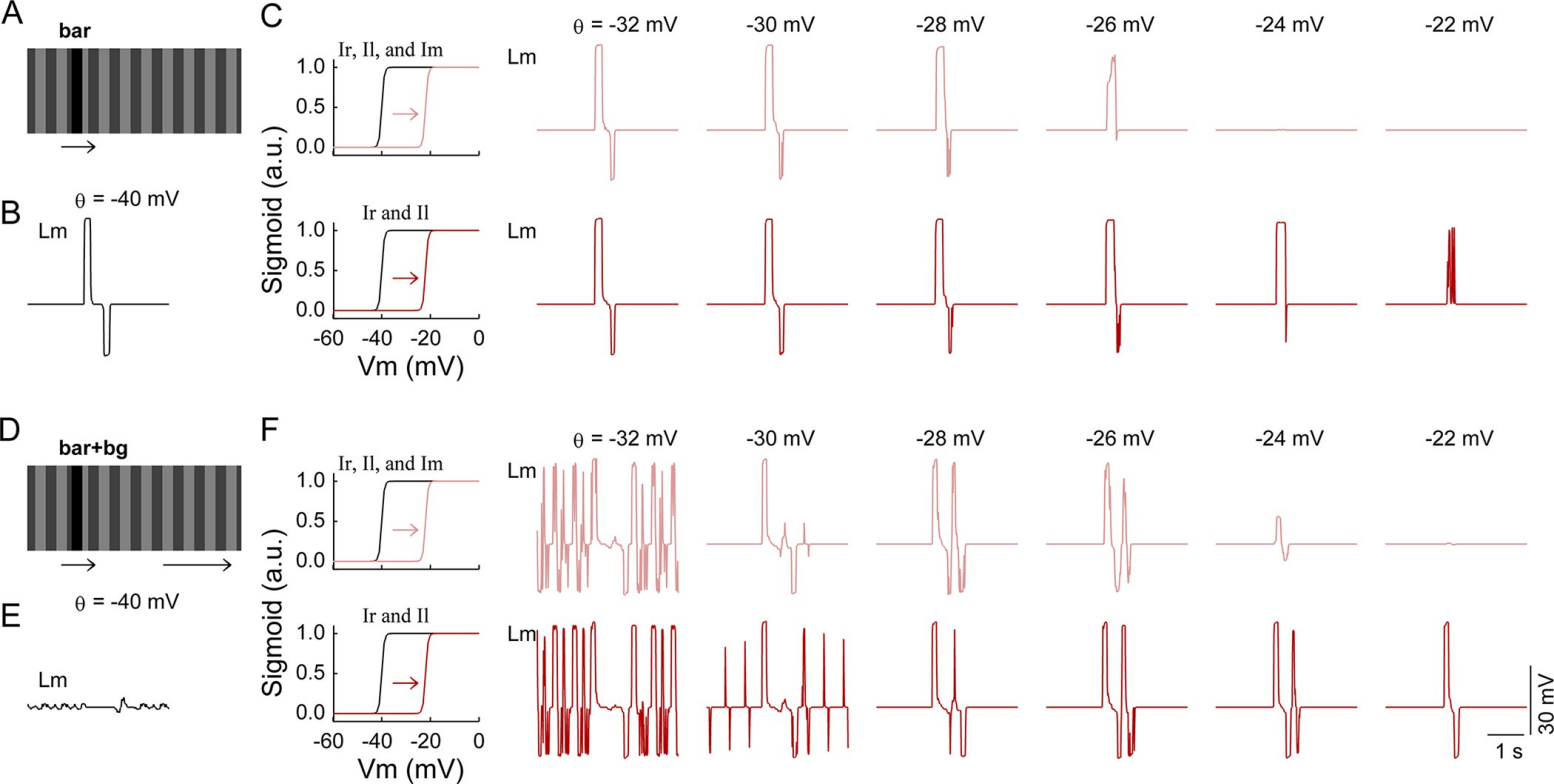
Effect of the modulated half-activation voltage on the responses of the units in the Lm module. (**A**) Same stimulus as ‘bar’ in Fig 8A. (**B**) Same data as shown in the panel located in the bottom row and the 1st column of Fig 8B. (**C**) Stimulus-evoked membrane potentials in Lm (2nd to 7th columns). Only the responses of the centrally located unit in Lm are displayed. By shifting the half-activation voltage *θ* to less negative values (indicated on the top of each column), the sigmoid function (black curves, 1st column) was shifted to the right (pink and red curves, 1st column). The module names to which the mimicked octopamine modulation process was applied are marked on the top of the 1st column. (**D**) Same stimulus as ‘bar+bg’ in Fig 8A. (**E**) Same data as shown in the panel located in the bottom row and the 3rd column of Fig 8B. (**F**) Same as (C) except that the simulation was performed with the ‘bar+bg’ stimulus in (D). The scale bars in the bottom right corner apply to all the membrane potentials. Except for *θ*, all parameter values were the same as those used to produce the results shown in Fig 8.

We next mimicked the application of an octopamine agonist to the model. The octopamine agonist increased the response gains in neurons ranging from medulla columnar neurons via T4/T5 neurons to lobula plate tangential cells [44, 45]. The increase was stronger at higher temporal frequencies instead of being uniform across the tested temporal frequencies of the grating stimuli [45–48]. Active locomotive states, thought to be related to increased endogenous octopamine release, also enhanced the response gains in LPLCs, with larger gains obtained at higher stimulus speeds [46, 49–52]. Based on these data, our model hypothesized that one direct or concomitant effect of octopamine was a shift in the response sensitivity of the postsynaptic neurons to the more depolarized state of their presynaptic neurons. The activation function $1/\left(1+e^{\left(\theta -V(t)\right)/\beta }\right)$ describes how a neuron unit with a membrane potential *V*(*t*) contributes synaptic conductance to its postsynaptic units in the lobula network (see ‘Methods’). The half-activation voltage *θ* is the presynaptic voltage at which the activation function has the largest sensitivity. We shifted the parameter *θ* of the units in the Ir and Il modules from –40 mV to less negative values to mimic the hypothesized octopamine effect (**Fig 9C and 9F**, leftmost panels in the lower rows).

We repeated the simulation above with a systematically shifted *θ* value. Under the ‘bar+bg’ stimulus condition, *θ* value shifts that were too small (e.g., *θ* ≤ –30 mV) or too large (e.g., *θ* ≥–20 mV) induced a randomly activated or nearly silent state, respectively, in the LC15-like units. Once the *θ* value fell within a specific range around [–28 mV, –22 mV] (called the “optimal modulation range” below), the responses to the dark bar in the LC15-like units were restored (**Figs 9F**, lower row and **8B**, 4th column). Their responses were not significantly altered under the ‘bar’ stimulus condition (**Figs 9C**, lower row and **8B**, 2nd column). The effect of octopamine modulation with only *θ* = –28 mV is displayed in **Fig 8B** for clarity. The responses of the LC15-like units were qualitatively similar to those recorded in LC15 with an application of the octopamine agonist [15] if their hyperpolarization component was ignored. We further evaluated the F-measure. The mimicked octopamine effect restored the capacity of the Ir module to discriminate the dark bar from its moving background, as shown by the example in **Fig 8C** (red versus black curves, right panel).

Moreover, we examined the effect of a larger scope of octopamine modulation. Specifically, the half-activation voltage *θ* of the units, not only in Ir and Il but also in their postsynaptic Im, were simultaneously shifted to less negative values. By repeating the above simulations, we found that the results were qualitatively similar under two spatial scope conditions of octopamine modulation (**Fig 9C and 9F**, upper rows). The only difference is that the optimal modulation range was changed from approximately [–28 mV, –22 mV] to approximately [–30 mV, –26 mV].

**Fig 10:**
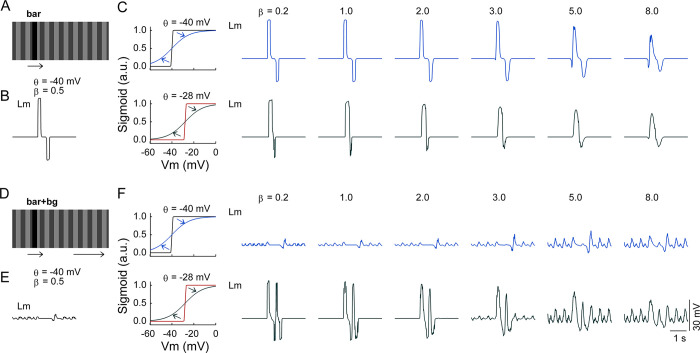
Effect of the modulated steepness on the responses of the units in the Lm module. (**A**) and (**B**) are the same as Fig 9A and 9B, respectively. (**C**) Stimulus-evoked membrane potentials in Lm (2nd to 7th columns). Only the responses of the centrally located unit in Lm are displayed. The parameter *β* was set as indicated on the top of each column, accordingly changing the sigmoid function’s steepness (4 curves in the 1st column). The half-activation voltage *θ* was fixed at –40 mV (upper row) or –28 mV (lower row). The scope of parameter modulation covered the Ir, Il, and Im modules. (**D**) and (**E**) are the same as Fig 9D and 9E, respectively. (**F**) Same as (C) except that the simulation was performed with the ‘bar+bg’ stimulus in (D). The scale bars in the bottom right corner apply to all the membrane potentials. Except for *β* and *θ*, all parameter values were the same as those used to produce the results shown in Fig 8.

Another critical parameter of the sigmoid activation function is the steepness *β*, which describes how rapidly a neuron unit with a membrane potential *V*(*t*) contributes synaptic conductance to its postsynaptic units. We further repeated the simulations above with elevated (i.e., decreased steepness) or lowered (i.e., increased steepness) *β* values (**Fig 10**). The activation function with an increased steepness was found to facilitate the responses of the lobula units (**Fig 10C**, upper row), but the increased steepness alone was unable to rescue the responses to the bar in the LC15-like units suppressed by the background motion (**Fig 10F**, upper row). To produce an efficient rescue, the increased steepness must be combined with a shifted half-activation voltage *θ* to less negative values (**Fig 10C and 10F**, lower rows).

Taken together, the model simulations qualitatively reproduced the experimental observations in the visually evoked LC15 responses with and without octopamine agonist application [30,31]. The simulations suggest that the response properties of the LC15 cells could be due to an octopamine-induced shift of the input–output characteristics in their presynaptic input cells.

### Figure-ground discrimination is robust to natural scene variability

**Fig 11:**
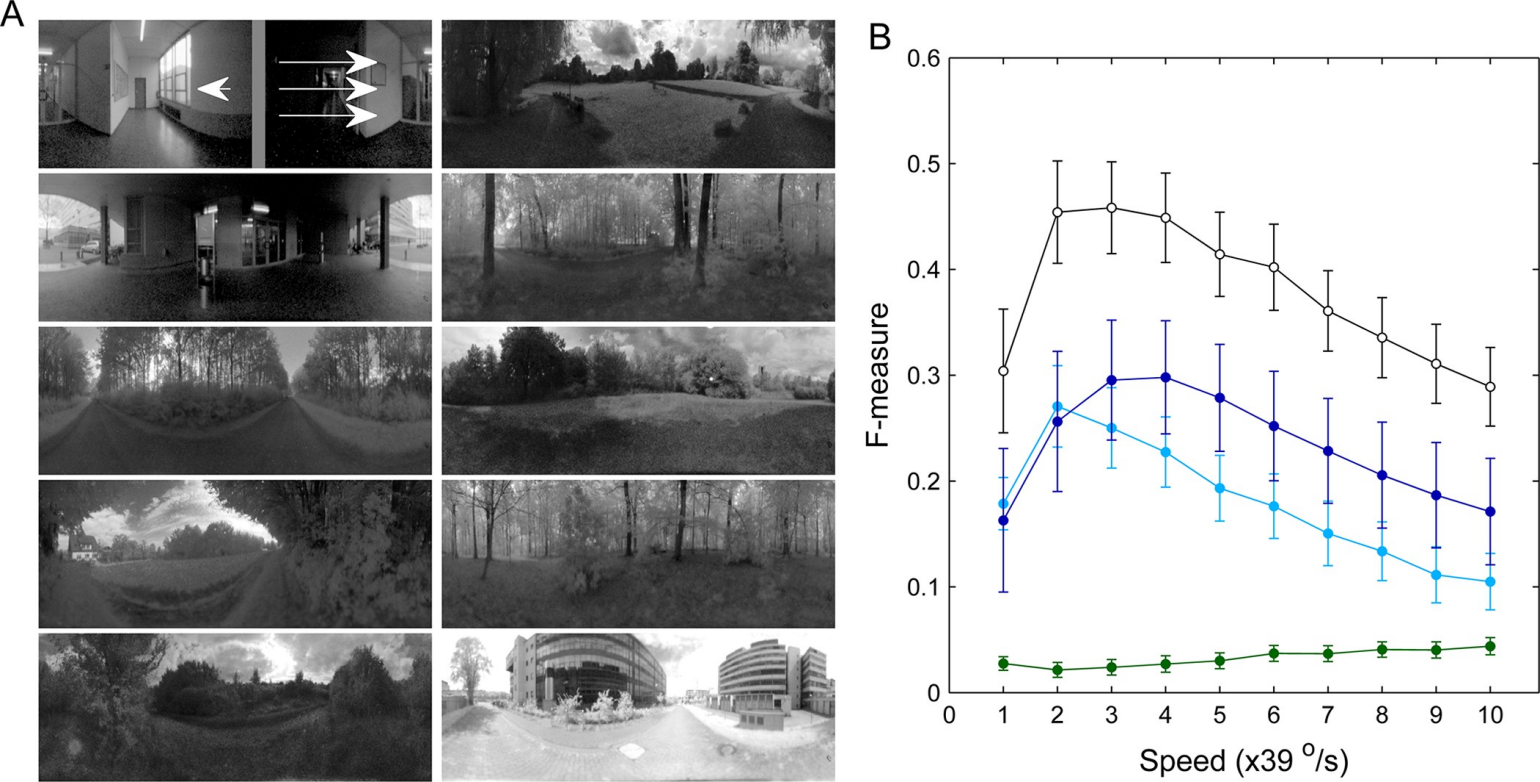
Figure-ground discrimination results produced by the model in the case of cluttered natural scenes. (**A**) Schematic illustration of the synthesized stimuli covering a full 360° azimuth and a 97° elevation. The foreground was a gray bar with a width of 15° and 0.5 luminance intensity, as illustrated in the panel located in the 1st row and 1st column, in which the arrowhead and three arrows express the movement directions of the bar and the background, respectively. The image background moving in reverse shared the same speed with the bar. The *C*_RMS_ levels of the images in the 1st column from top to bottom are 5.74, 3.89, 2.10, 1.59, and 1.28, respectively. The *C*_RMS_ levels of the images in the 2nd column from top to bottom are 1.19, 0.92, 0.88, 0.69, and 0.43, respectively. (**B**) F-measures evaluated at three stages along the pathway from the EMD array to the Il module: the output of the EMD array (green curve), the input of Il (light blue curve), and the output of Il (dark blue curve and black curve). The F-measures were averaged across all 10 conditions of the background scene for each stimulus speed. Error bars indicate the standard deviations. The synaptic weights of the projections from the EMD array to the postsynaptic units were set as *α*^*EMD*→*lo*^ = 100 (all curves except the black one) and 200 (the black curve), respectively.

Natural scenes vary in texture, contrast, and luminance. This variability affects the visual motion estimation results produced by the EMD array, making figure-ground discrimination more challenging than that with laboratory-designed visual stimuli. To determine whether the present model robustly detects figures (or objects) embedded in cluttered natural scenes, we created a set of synthetic stimuli by superimposing a moving gray bar upon high-dynamic-range (HDR) images moving in reverse (**Fig 11A**). The HDR images were obtained from a database recorded in diverse environments by Meyer et al. [53]. Image contrast level was quantified by the RMS contrast *C*_RMS_, which was calculated by dividing the global standard deviation by the global mean luminance of each original HDR scene image (‘Methods’). Almost all the images in the database [53] have a *C*_RMS_ ranging from 0.4 to 5.7, being similar to the *C*_RMS_ span of HDR images in another database [54]. Without loss of generality, ten HDR images with various lighting and spatial clutter conditions, whose *C*_RMS_ levels covered the entire range [0.4, 5.7], were used as the background of the synthesized stimuli (**Fig 11A**). Before being fed to the model, the HDR images were preprocessed by Lipetz [55] or Naka and Rushton [56] transformation (‘Methods’), mimicking light adaptation in the retina, which needs to operate in an extremely broad range of light intensities.

We ran the model by focusing on the pathway connecting the EMD array with its postsynaptic module Il, as this module yielded depolarization responses to the bar moving to the left. The simulations show that F-measures were considerably low at the output stage of the EMD array (less than 0.05), corresponding to an impossible figure-ground discrimination scenario (green curve, **Fig 11B**). The F-measures were largely improved downstream of the EMD array. The improvement achieved at the output stage of the Il module (after spatial-temporal smoothing of the visual motion) was larger than that at its input stage (after spatial smoothing alone) (dark blue curve versus light blue curve, **Fig 11B**).

However, compared with those obtained using richly textured laboratory-designed stimuli (e.g., **Fig 4B and 4D**), the F-measures were substantially lowered at all three stages due to the much sparser EMD responses caused by natural scenes. The sparser EMD responses should lower the conductances *g*^+^(*t*) and *g*^−^(*t*) from the EMD array to its postsynaptic units. According to Eq (2), the synaptic current *I*^syn^ would be lowered and lead to impaired figure-ground discrimination. To test this possibility, we repeated the above simulation by increasing the synaptic weight *α* to compensate for the decrease in the synaptic current. The F-measure was indeed boosted at the output stage of the Il module (**Fig 11B**, black curve). The boosting effect saturated for *α* values that were approximately greater than 300. The modulation in parameter *α* mimics an adaptation and/or plasticity mechanism, which should be necessary for fly motion vision to process natural scene variability.

In summary, the model is capable of computing figure-ground discrimination solutions in the case of natural scene variability.

## Discussion

### EMD model

The EMD-lobula network model is robust to small perturbations in the parameters of the lobula network and stimulus patterns, as shown in the Results section. Here, we focus on the reasonability of the EMD model that has not yet been fully discussed.

We used the 2-Quadrant-Detector version of the EMD model proposed by Eichner et al. [39] (called the 2-Quadrant-Detector model below). However, significant progress has been made in models of the fly EMD over the past decade. For instance, individual EMDs have been revealed to be three-armed rather than two-armed in both the ON and OFF motion pathways in *Drosophila* [57–59]. The subtraction stage between mirror-symmetrical half-detectors of the Hassenstein–Reichardt model does not occur on T4/T5 cells but on tangential cells through inhibitory inputs from lobula plate LPi interneurons [60]. We chose to use the 2-Quadrant-Detector model, which has a two-armed structure, in the present study for a few reasons.

First, the 2-Quadrant-Detector model was based on the full Hassenstein–Reichardt model after the subtraction stage, which yields a high degree of directional selectivity that is characteristic of three-armed EMD model. The full Hassenstein–Reichardt model deserves credit for cancelling out direction-unspecific components of the response, enabling the EMD output to be highly selective for the motion direction [61]. Direction-unspecific response components may arise even from brightness changes in the inputs to both half-detectors, which are unrelated to motion. The subtraction stage, however, generates EMD responses with opposite signs. By using the 2-Quadrant-Detector model [39], we do not mean in any sense that there exists a local motion opponency in T4/T5 cells. The present model treated the EMD output with opposite signs as the responses of two groups of T4/T5 cells (one for each horizontal direction). The positive responses of the EMD array were assumed to induce excitatory and inhibitory inputs to the Ir and Il modules, respectively, and vice versa for negative responses (see ‘Methods’). The treatment is computationally equivalent to an application of a mathematical transformation, by which the negative sign is usually removed from the output of the full Hassenstein–Reichardt model [e.g., 62].

Second, the results of our EMD-lobula network are quantitatively robust to small perturbations in the parameters of the 2-Quadrant-Detector model, such as the time constants for the high-pass and low-pass filters, the cutoff for the ON and OFF rectifiers, and the proportion of the DC input. Qualitatively, the presence or absence of the DC input has no practical impact on the results of our EMD-lobula network.

The ON and OFF motion pathways in flies are similar in their function, although significant differences have been revealed between the two pathways [63,64]. Our model retinotopically sums the ON and OFF EMD arrays for simplicity. We blocked the ON or OFF pathway of the 2-Quadrant-Detector model and repeated the simulations with the first type of stimuli. The conclusions of the present study were qualitatively unchanged by the blockage, except for a mild decrease in the F-measure. This indicates that the EMD-lobula network does not depend on whether the ON and OFF EMD pathways are combined. Future studies are expected to provide insights into the differences in figure-ground discrimination between the two pathways.

### Testable predictions

A prediction of the model is that T4/T5 cells are the major, if not exclusive, source of the directional motion sensitivity of the lobula. Specifically, the directional motion sensitivity of the lobula is predicted to originate mainly from T4/T5 cells via neurons that include at least the translobula-plate neurons (e.g., Tlp1, Tlp2, Tlp3, and Tlp4) and thus are called “Tlp-interneurons” below. The translobula-plate neurons connect different layers of the lobula plate with the lobula, whose morphological characteristics suggest that they pass information mainly from the lobula plate to the lobula [20].

Lobula plate-lobula neurons, e.g., LPLC1 and LPLC2, have already been identified to be directly driven by T4/T5 cells and exhibit apparent presynaptic sites in the lobula [28,65–67]. LPLC1 and LPLC2 present their strongest responses to impending collision-related stimuli [28,31,66,68]. We speculate that LPLC1 and LPLC2 spatiotemporally smooth their inputs from T4/T5 cells themselves, which should be critical for these cells to detect approaching targets.

Considering that a large majority of the medulla outputs project to the lobula [20], does another non-T4/T5 circuit upstream of the lobula compute local directional motion signals? Do the lobula VPNs themselves compute the motion signals based on presynaptic inputs obtained from the medulla? Our model does not exclude such possibilities. For example, columnar T2 and T3 neurons that are highly sensitive to small moving objects were recently identified to be presynaptic to LC11, suggesting that LC11 may inherit its motion sensitivity to small objects from the T2/T3 inputs in the medulla [69]. Despite such possibilities, our model predicts that the non-T4/T5 sources of lobula motion sensitivity are limited in numbers.

Second, the model predicts that downstream of the Tlp-interneurons, the lobula possesses parallel pathways for visual feature extraction. These pathways may differ in their receptive field structures and/or directional preferences. For example, there should be two parallel pathways that share an elongated receptive field with a specific orientation but separately prefer rightward and leftward movements. They separately address visual motion-based object detection and project to the same motor center, forming parallel and winner-take-all sensorimotor pathways.

Third, the model predicts that octopamine induces a shift in the input–output characteristics of the presynaptic input cells of the feature extractor neurons in the lobula (e.g., LC15), causing the lobula circuit to compute figure-ground discrimination in a state-dependent manner. For instance, translobula-plate neurons should display such shifted input–output characteristics once they experience an octopamine agonist application.

### Comparison with previous models

A previous model proposed by Li et al. [70] suggested that motion adaptation facilitates the segregation of a nearby object from its cluttered background. According to Koenderink and van Doorn [71], the nearby object induced stronger responses in the EMD array than the background did when only translational component of self-motion was considered. Motion adaptation further amplifies the difference between the responses induced by the object and the background, causing the nearby object segregated at the output stage of the EMD array [70]. It took several seconds for their model to adapt to motion changes, and the model efficiency decreased once the stimulus velocity became constant. This contrasts with our model, in which the spatiotemporal smoothing operation is always inherent in the lobula network.

Theta motion is visible to flies [32,72]. An EMD array cannot detect the motion direction of ‘Theta Figure’ (see the stimulus in the rightmost panel in **Fig 3A**). A model proposed by Zanker and colleague consisting of two layers of EMDs [33–35], however, successfully extracted the ‘Theta Figure’ direction. The most fundamental difference between the two models lies in the fact that the second layer had no directional selectivity in their model. In our model, the output of the first layer is processed by two lobula modules whose directional selectivities are opposite of each other. Our model easily discriminates a ‘Theta Figure in Moving Background’, in which the theta figure shares exactly the same motion as its background (**Fig 5B1 and 5B2**). Detecting the direction of the ‘Theta Figure in Moving Background’, however, should be a challenge for their model.

Two bioinspired methods for small obstacle detection were recently proposed and shown to be applicable to small aerial platforms where constraints in weight and computational power are important [73]. This is different from our model that has not yet been designed for an aerial platform whose trajectory needs to be controlled. The two methods [73] were inspired by the emergence mechanism of target selectivity in FD cells [18–19] and the object size-dependent detection mechanism of an EMD array in the second stage [74–75]. Their methods mainly relied on the difference between the magnitude of local parallax vectors induced by a nearby small obstacle and its far background in the optic flow field. The obstacle was extracted as a high spatial-frequency component of the optic flow field [73]. Their methods have advantages in extracting the relative range or nearness of the nearest obstacle. In contrast, our model mainly utilizes the directional difference between the object and its background. Although our model can readily discriminate near obstacles from far backgrounds, it would be unsuitable to discriminate obstacles that are close to each other. This is because spatiotemporal smoothing will weaken, to varying degrees, the dependence on distance of the translation-induced component of local parallax vectors. On the other hand, their methods operated only in the azimuthal direction and were, thus, not designed for extracting the shape of moving objects. In contrast, our model seeks to extract an entire figure as accurately as possible. In addition, their methods were essentially optimal for narrow obstacles, whereas our model has no size restrictions on target width.

In conclusion, we develop a bioinspired EMD-lobula network model to investigate how an accurate figure-ground discrimination is able to be achieved with correlation-type motion detectors that cannot measure true velocities. Our simulations find that heavily fluctuating output of an EMD array can be spatially and temporally integrated and, thus, naturally smoothed out by its downstream lobula network that consists of parallel pathways with distinct directional selectivity. The visual motion smoothing enables a robust segmentation of moving figures from the background. Furthermore, the model qualitatively reproduces the experimental observations in the visually evoked response characteristics of one type of LC cell (i.e., LC15 cells). The results suggest that the lobula is involved in visual motion-based target detection. The results could also be inspiring for motion detection in machine vision systems that rely only on a single camera.

## Methods

### Visual stimulus inputs

Three types of visual stimuli were used in the study. The first type (used for simulations, the results of which are shown in **Figs 2–6**) was created with a resolution of 0.33° per pixel in a manner similar to that of Tammero and Dickinson [76]. The frame images covered 180° in azimuth and 90° in elevation. Both the figure and background consisted of randomly distributed black and white dots, making the figure invisible unless it was moving relative to the background. The relative luminance of any dots can be expressed as *I*_max_ = *I*_0_+Δ*I* and *I*_min_ = *I*_0_+Δ*I*, respectively, where *I*_0_ is the average luminance and Δ*I* is the luminance difference. By fixing *I*_0_ = 0.5 and modulating Δ*I*, we set the stimulus contrast based on the Michelson contrast (*I*_max_−*I*_min_)/(*I*_max_+*I*_min_). Unless otherwise specified below, a dot size of 8 × 8 pixels (i.e., 2.6° × 2.6°) and a contrast of 80% were established for this stimulus type.

The second stimulus type (used for simulations, the results of which are shown in **Figs 7–10**) was designed to cover a visual field of 180° in azimuth and 70° in elevation, imitating the stimuli in Städele et al. [30]. The image resolution was 0.33° per pixel. In **Fig 7**, the relative luminance levels of the foreground and background in the ‘small object’ and ‘bar’ stimuli were set as 0.25 and 0.75, respectively. The relative luminance of the ‘wide-field’ square grating was periodically set as 0.25 and 0.75. In **Figs 8–10**, the stimuli were dark bars (with 0 luminance) moving over a square grating with a Michelson contrast of 33%, whose relative luminance was periodically set as 0.25 and 0.5. For both the first and second types of stimuli, the relative luminance was normalized to 0 for darkest black and 1 for lightest white. Absolute illumination has been suggested to affect the dynamic ranges of motion-sensitive neurons [77], but the effect of absolute illumination on figure-ground discrimination is beyond the scope of our model.

The third stimulus type (used for simulations, the results of which are shown in **Fig 11**) was synthesized by superimposing a uniform gray bar on natural scenes obtained from an image database recorded in diverse environments [53]. The scene images were in HDR format, covering 360° in azimuth and 97° in elevation with a resolution of 0.39° per pixel.

The HDR images varied greatly in mean luminance. We quantified their contrast by calculating the RMS contrast (denoted as C_RMS_). It is defined as *C*_RMS_ = *I*_SD_/*I*_mean_, where *I*_SD_ and *I*_mean_ are the standard deviation and mean of the pixel luminance intensities in each original image, respectively [54,78]. Dividing by the global mean luminance enables *C*_RMS_ to not be dependent on image luminance.

We applied Lipetz [55] or Naka and Rushton [56] transformation to the luminance intensity *I* of the HDR images before feeding them to the EMD array. This mimics light adaptation in the retina via the nonlinear response characteristic of photoreceptors:

$$I^{\prime }=\frac{I^{\mu }}{I^{\mu }+I_{\mathrm{mean}}^{\mu }}$$ (4)

where *μ* was set to 0.7 [79,80]. Thus, the luminance intensity *I*′, being actually fed to the EMD array, ranged from 0 to a value slightly smaller than 1.

For all three types of stimuli, each frame was blurred by a 2D Gaussian low-pass filter containing 12 × 12 pixels with a standard deviation of *σ* = 3.5 pixels and then downsampled by a factor of *η* = 6 pixels along both dimensions to simulate the properties of the fly peripheral visual system. The photoreceptor acceptance angle and the interommatidial angle of the modeled optics can be described as $\Delta \rho =2\sqrt{2\ln2}\sigma$ (full width at half maximum) and Δϕ = *η*, respectively. According to the resolution (in degrees per pixel) given above, Δρ = 2.7° and Δϕ = 2.0° under the first and second types of stimulus conditions and Δρ = 3.2° and Δϕ = 2.3° under the third type of stimulus condition. The preprocessing step transformed the input frames into matrices of time-varying intensities, as illustrated by one example in the middle panel of **Fig 2A**. The intensity matrices were sequentially fed into the EMD array to detect directional motion.

### EMD array

The EMD array was developed with each unit receiving two inputs separated in visual space. The individual EMD units comprised parallel ON and OFF detector subunits, whose detailed structure is exactly the same as shown in **Fig 4A** in the paper by Eichner et al. [39]. The visual input was first processed by a first-order high-pass filter with a time constant of *τ*_HP_ = 250 ms (except for 10% of the original signal that was allowed to pass unfiltered). The filtered and DC components were added and then fed into two parallel half-wave rectifiers, one forming the ON pathway and the other forming the OFF pathway. The cutoffs for the ON and OFF rectifiers were set at 0 and 0.05 of the incoming relative luminance signal, respectively. First-order low-pass filters with *τ*_LP_ = 50 ms were adopted in both the ON and OFF subunits. The subtraction stage of the detector subunits was modeled to prefer rightward motion, i.e., to yield a positive (negative) output for rightward (leftward) motion.

The ON and OFF detector subunits did not provide outputs unless the absolute amplitude of their responses reached a threshold of *thr* = 0.002. This nonlinearity removed random noises with very small amplitudes, but the presence or absence of the parameter *thr* had no practical impact on our results. To further simplify the problem, the outputs of the two EMD arrays in the ON and OFF pathways were retinotopically added to form the final output of the ON+OFF EMD array, which was then projected to the Ir and Il modules.

### Lobula network

The lobula network started from Ir and Il, whose neuron units received input from the ON+OFF EMD array but with opposite Gaussian receptive fields (**Fig 1A and 1B1**, left versus right panel). Unless otherwise specified, each receptive field was set to be a square area with a side length of 7 EMD units, which was equivalent to 14° × 14° for the first stimulus type. The outputs of the Ir and Il modules were retinotopically summed and projected to the Im module, making the units in Im have no directional selectivity. The receptive field of Im on the Ir and Il modules was set as a square area with a side length of 3 lobula units. Specifically, the receptive field was modeled as a 2D Gaussian function, whose specific matrix form was [0.0 0.1 0.0; 0.1 0.6 0.1; 0.0 0.1 0.0] with three rows separated by the semicolons (**Fig 1B2**).

The Ir, Il, and Im modules are projected to the Lr, Ll, and Lm modules in the last layer, respectively. Lr, Ll, and Lm inherited directional selectivity from their respective presynaptic modules. The receptive fields of their units were set as 3 × 3 Prewitt filters, mimicking edge detectors along the vertical direction. The Prewitt filters had a matrix form with 0.05 × [1 0–1; 1 0–1; 1 0–1], whose three rows are separated by the semicolons (**Fig 1B3**). Prewitt filters were selected due to their efficiency and simplicity. More functions, such as texture analysis and shape recognition, were not simulated in Lr, Ll, and Lm; otherwise, Gabor filters, not Prewitt filters, should be used.

A sigmoid function $1/\left(1+e^{\left(\theta -V_{i,j}(t)\right)/\beta }\right)$ was used as the activation function of the lobula units, where parameters *θ* and *β* are the half-activation voltage and steepness, respectively. We set *θ* = −40 mV and *β* = 0.5 unless otherwise specified.

Driven by visual stimuli with a 100 Hz refresh frequency, the synaptic conductance matrices of Ir and Il were updated every 10 ms, while all the units in the lobula network were integrated with a time step of 0.4 ms. Synaptic currents to individual lobula units were proportional to parameter *α* according to Eq (2), which represents a synaptic weight. Values of the parameter *α* were chosen such that the induced activity in postsynaptic neurons was moderate under the condition that both receptive field size and stimulus parameter values were intermediate (see **Fig 6** for ranges of receptive field size and stimulus parameter values). Unless otherwise specified, the parameter was set as *α*^*EMD*→*lo*^ = 150 if the given unit was postsynaptic to the EMD array and *α*^*lo*→*lo*^ = 20 if the unit was postsynaptic to one of the modules in the lobula network.

### Quantitative evaluation of figure-ground discrimination by the F-measure

We used the F-measure to evaluate the degree of correctness exhibited by figure-ground discrimination because the F-measure is an evaluation metric for assessing classifier performance in a two-class classification problem. The F-measure is especially convenient for evaluating motion detection methods [81].

The result of figure-ground discrimination (i.e., the output of the Ir or Il module) was transformed into a series of binary images by setting a 50% maximum segmentation threshold (e.g., **Fig 4A and 4C**, top rows), in which white and black pixels represented foreground and background pixels, respectively. Based on the ground truth of each frame, we could recover four quantities: true positives (*TP*), false positives (*FP*), true negatives (*TN*) and false negatives (*FN*). *TP* and *FP* are the numbers of foreground pixels correctly and incorrectly classified, respectively. *TN* and *FN* are the numbers of background pixels correctly and incorrectly classified, respectively.

The F-measure is defined by combining two other metrics called *precision* and *recall*. As shown in Eq (3), it is defined as *F-measure* **=** (2 × *precision* × *recall*)/(*precision* + *recall*) = 2*TP*/(2*TP* + *FP* + *FN*), where *precision* = *TP*/(*TP* + *FP*) and *recall* = *TP*/(*TP* + *FN*). The F-measure is a value between 0.0 for the worst case (failed figure-ground discrimination) and 1.0 for the best case (perfect discrimination).

The F-measures at the output stage of the EMD array were evaluated based on the output of the ON+OFF EMD array. The F-measures at the input stage of the Ir or Il module were evaluated based on their synaptic conductance matrices, which were calculated by convolving the output of the ON+OFF EMD array with the receptive field function of the corresponding module. The data at these intermediate stages were preprocessed into a series of binary images, to which the *F-measure* formula was then applied. To ensure an objective evaluation, we took into account both the positive and negative components of the data at each of the intermediate stages.

### Network simulations

Simulation programs were written in the MATLAB programming language (R2013b) and run on a Dell Precision T7810 workstation. The EMD array was a discrete-time system with a time step of 10 ms, while the lobula network was numerically integrated as a continuous-time system using the fourth-order Runge–Kutta method with a time step of 0.4 ms. To link up different time scales, the EMD array was updated every 10 ms, during which the lobula modules were successively integrated for 25 time steps.

## Supporting information

S1 Video A textured square moving on a similarly textured background. (MP4)

S2 Video A small dot textured theta figure moving together with a similarly textured background. (MP4)

S3 Video A large dot textured theta figure moving together with a similarly textured background. (MP4)
